# Postoperative Pain of Pediatric Patients Undergoing Dental Treatment under General Anesthesia Visiting a General Hospital: A Cross-Sectional Study

**DOI:** 10.3390/children10040671

**Published:** 2023-03-31

**Authors:** Ateet Kakti, Reema Khalid Abumelha, Asmaa Mansour Alajmi, Lamis Khalid Dagriri, Lamia Abdullah Alkodari, Mohammed. J. Fares, Marco Cicciù, Giuseppe Minervini

**Affiliations:** 1 Department of Preventive Dentistry, College of Dentistry, Riyadh Elm University, Riyadh 13244, Saudi Arabia; 2 College of Dentistry, Riyadh Elm University, Riyadh 13244, Saudi Arabia; 3 October University for Modern Sciences and Arts, 6th October City 12573, Egypt; 4 Department of General Surgery and Medical-Surgical Specialties, School of Dentistry, University of Catania, 95131 Catania, Italy; 5 Multidisciplinary Department of Medical-Surgical and Dental Specialties, University of Campania, Luigi Vanvitelli, 80138 Naples, Italy

**Keywords:** postoperative pain, dental general anesthesia, children, pediatric dentistry, special care

## Abstract

Dental general anesthesia (GA) is a day-stay procedure and is a suitable choice for complicated cases. It is undertaken in a controlled hospital setting that ensures the quality, safety, efficacy, and efficiency of dental treatment. The purpose of this study is to determine the prevalence, severity, duration, and factors related to the occurrence of postoperative discomfort in young children following GA in a general hospital. This study includes a minimum sample size of 23 children that were undergoing GA over a 1-month period. Informed consent was obtained from the parent prior to the treatment. A preoperative questionnaire via the Survey Monkey program was used for the purposes of recording the responses of the survey population. All data related to the immediate postoperative period while the child was in the post-anesthetic recovery room (PAR) was collected and assessed by one of the investigators using the Face, Legs, Activity, Cry, and Consolability (FLACC) pain assessment scale. Postoperative data was gathered using the Dental Discomfort Questionnaire (DDQ-8) and was performed by phone 3 days after the GA procedure. The participating 23 children ranged from 4 to 9 years old (mean 5.43 ± 1.53). A total of 65.2% were girls and 34.8% were boys, with 30.4% experiencing a recent history of pain.

## 1. Introduction 

Delivering treatment to children can be difficult, particularly where extensive treatment is required [1,2,3,4]. In addition, it is known that oral comprehensive treatment of children can result in different physical and psychological trauma [5]. There are many different behavioral and therapeutic approaches in managing extensive dental issues in young children. Dental fear in children has been recognized in many countries as a public health problem. GA is preferred as an alternative when undertaking dental treatment on children for this reason [6,7]. One of the principal approaches is to use general anesthesia (GA) during comprehensive oral treatment in children who are very young in age, or who suffer from extreme anxiety and/or physical or mental disabilities [8,9].

Pediatric patients often experience fear and anxiety when undergoing local anesthesia, which can lead to increased pain perception. Various attempts have been made to reduce pain during local anesthesia in pediatric patients, including distraction techniques, topical anesthetics, and buffering of the anesthetic solution. Nitrous oxide, also known as laughing gas, has also been used as a form of analgesia for pediatric patients during local anesthesia procedures [10,11,12,13,14,15,16,17,18]. Nitrous oxide is a colorless, odorless gas that is inhaled through a mask and provides a mild form of sedation and pain relief [10,11,12,13,14,15,16,17,18]. It is commonly used in combination with other forms of pain management such as local anesthesia injections, in order to reduce discomfort and anxiety in pediatric patients [10,11,12,13,14,15,16,17,18]. Nitrous oxide has been found to be safe and effective for use in children undergoing various dental and medical procedures. Dental GA is a day-stay procedure and is a suitable choice for complicated cases. It is performed in a controlled hospital [10,11,12,13,14,15,16,17,18] setting that ensures the quality, safety, efficacy, and efficiency of dental treatment [8,19,20,21,22] Another benefit of GA is that all treatments can be completed in one visit, with less anxiety for both the patient and the parents, and more comfort for the dentist [9]. Its usage is quite practical for dental institutions, and minimizes the economic burden for the children’s families [23]. It has been documented that 60% of pediatric dentists are utilizing GA to treat pediatric patients. However, it has been reported that the main restricting factor for using GA is the safety of the children in whom it is administered [24]. It involves complications including pain, which is the most common issue, as well as bleeding, nasal and mouth discomfort [23].

Pain is an unpleasant sensory and emotional experience associated with, or described in terms of, actual or potential tissue damage [25]. It is subjective and differs from person to person, and the gold standard for judging pain is by self-reporting [26,27,28,29]. Currently, the practice shows that parents and health care providers tend to underrate the child’s pain when compared with the child’s self-report. This is due to the incapability of young children to verbalize, understand, and express their experience, in addition to adults’ incapability to identify and detect signs of pain in their young children [30].

To assess the patient’s safety with regards to morbidity after comprehensive dental treatment, international studies are quoted due to insufficient local data on this topic [8]. In addition, the limited local data on postoperative morbidity following pediatric dental GA procedures is the main purpose of this study.

## 2. Materials and Methods

This was a prospective observational study that included a minimum sample size of 23 children who underwent GA at REU hospitals in Riyadh, Saudi Arabia over a 1-month period. All children were seen preoperatively at the Department of Pediatric Dentistry at REU. The Institutional Review Board at Riyadh Elm University (REU), Riyadh, Saudi Arabia authorized the protocol, which was carried out in accordance with the Helsinki Declaration (Protocol number: “FUGRP/2021/215/394/385”, Date: 23 December 2022). The written informed consent for involvement was provided by each patient and their guardians. Informed consent was obtained from the parent prior to data collection.

### 2.1. Inclusion Criteria

1. Healthy (American Society of Anesthesiologists status I).
2. Aged between 4 and 9 years old.
3. Communicate well in Arabic.
4. Require oral comprehensive treatment involving multiple dental procedures, for example: pulpotomies, pulpectomies, and extractions of at least one tooth.

### 2.2. Exclusion Criteria

1. Developmentally delayed or congenitally impaired children
2. Born prematurely (defined as 37 weeks gestational age)
3. Currently using psychotropic medications
4. Using analgesics preoperatively on the day of the procedure

### 2.3. Data Collection

Sample calculation: A sample of 23 participants were recruited to yield an 80% power to detect the effect of this sample size at a *p* value of 0.05.

A preoperative questionnaire was used via the Survey Monkey program, which was sent via a link before the patient entered the GA appointment with an accompanying caregiver. The survey was composed of eight questions and contained information on demographic characteristics, past GA experience, recent use of medications for a dental issue, history of tooth discomfort, and the DDQ-8, which is an instrument to measure dental pain or discomfort in children. The range for the total score was from zero to 16; a score of three or higher was been determined to predict tooth-related discomfort in children (Table 1) [31].

**Figure 1:**
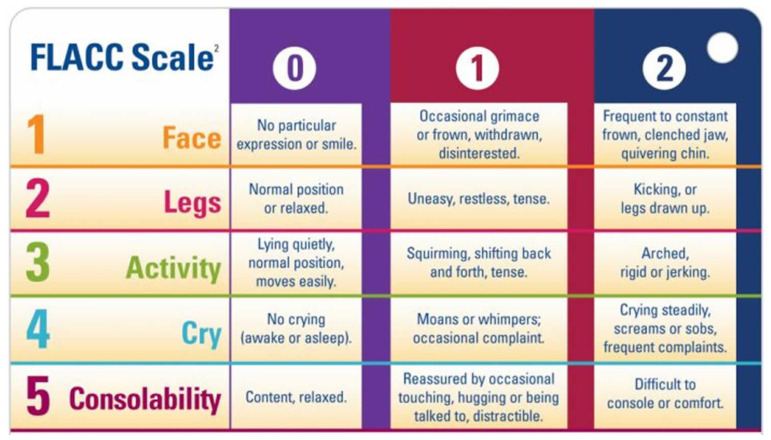
FLACC pain assessment tool.

All data related to the immediate postoperative period while the child was in the PAR was collected by one of the investigators. The child’s discomfort was assessed using the FLACC pain assessment scale. The range for the overall score was from 0 (no pain) to 10 (intense pain) (Figure 1) [32].

Postoperative data was gathered by an investigator using the DDQ-8, and an inquiry about the use of medications, the child’s capability to eat a regular diet, and the severity/duration of pain; this process was performed using a phone 3 days after the GA procedure. 

### 2.4. Dental General Anesthesia Protocol

A carer entered the dental surgical suite with the child for the induction of GA, which was administered intravenously. Three certified pediatric dentists supplied all of the dental care. For a few children who needed multiple extractions and crowns, infiltration local anesthesia (2% Scandicaine with epinephrine 1:100,000) was used. Children were extubated in the surgical suite by the anesthesiologist after receiving their treatment, and were then immediately admitted to the PAR under the care of the PAR nurse. Children were to be released 15–30 min after awakening, as long as they were steady. The carers received postoperative instructions.

### 2.5. Statistical Analysis

The normality of the data was assessed using the Shapiro–Wilks test, and it was found to be not normally distributed. Hence, non-parametric tests were applied to the data. A descriptive statistic of mean and standard deviation values was calculated for the continuous variables, and frequency distributions were obtained for the categorical data. Mann–Whitney U and Wilcoxon’s sign rank tests were applied to the data. A value of *p* < 0.05 was considered significant for all statistical purposes. All data was analyzed using SPSS version 25 (IBM-SPSS, 25 Armonk, NY, USA).

## 3. Results

The participating 23 children ranged from 4 to 9 years old (mean 5.43 ± 1.53). Of this sample size, 65.2% were girls and 34.8% were boys, with 30.4% experiencing the maximum pain. The descriptive data and characteristics of the study of the children are reported in Table 2. GA protocols were similar, but not exactly the same among the anesthetists. Furthermore, a comparison of the children’s preoperative and postoperative discomfort revealed a statically significant difference (Mann–Whitney U and Wilcoxon’s sign rank tests *p* < 0.05).

In Table 3, questions were asked of the caregivers whether the patient had a previous GA experience; 78.3% reported they had not. However, 21.7% of the children stated they had had a previous GA experience of some sort. In addition, a history of the chief complaint was taken in months, with a mean of 12.13 ± 11.65 (0.5–36). During the intraoperative a nasal intubation had been undertaken in all of the children, with the administration of LA in 19 of the patients, 82.6% using (Scandicaine 2% with epinephrine 1:100.000). Moreover, the total stay in the recovery room ranged between 15 and 40 min, with a mean of 27.83 ± 6.37 (15–40). Postoperative analgesics were prescribed and taken by 65.2% of the children. All accompanying caregivers preferred to be contacted for follow-up by phone within 3 days of the procedure. Alongside the DDQ8, yes and no questions were asked by one of the investigators about certain variables, such as whether the child was eating a regular diet or not (78.3% reported yes to this question), and if the child was on any current medications (78.3% answered no). All children had their preoperative and postoperative dental discomfort measured by the DDQ-8. Interestingly, question number 7 (is the child grabbing his/her cheeks during eating) shared the same highest and lowest scores in both pre-and post DDQ-8, with the highest being (pre: 23–56.5%), post (43–95.7%) (Table 4).

The FLACC scale was assessed immediately after waking up in the PAR. Most of the 23 patients (56.5%) expressed discomfort by crying steadily, including screams, sobs or frequent complaints, while a few of the expressions were evaluated by the face, legs, activity, cry, and consolability scale (21.7%) (Table 5). A comparison was undertaken between the two genders in regard to the pre DDQ-8, FLACC and post DDQ-8, which showed a significant decrease in the mean and a statically significant difference of *p* < 0.001 (Table 6). 

A different variable was used to indicate whether the child experienced discomfort during the dental treatment under GA in (Table 7). As reported in the regression model, it showed that none of these variables indicated or predicted whether there could be any future discomforts. An exception was the restoration category, which indicated discomfort (P-0.047). Furthermore, there was no correlation between the different variables with the DDQ8 (Table 8).

## 4. Discussion

This investigation was significant as it shed light on the factors that contribute to postoperative discomfort in young children, which can help improve their postoperative care after GA. Firstly, it provides valuable insights into the immediate and postoperative pain experienced by pediatric patients undergoing dental procedures under GA. The use of the FLACC pain assessment scale and the Dental Discomfort Questionnaire (DDQ-8) provide objective and subjective measures of pain, respectively, allowing for a more comprehensive understanding of the pain experienced by the children. Secondly, the study’s sample size of 23 children over a 1-month period is significant, as it provides a reasonable representation of the population being studied. This sample size also allows for statistical analysis and provides a basis for future studies and comparison with other similar studies. Thirdly, obtaining informed consent from the parent prior to treatment is an ethical requirement, and the use of a preoperative questionnaire via the Survey Monkey program ensures that the parent’s perspectives and concerns are taken into consideration. This shows that the study was conducted in an ethical and responsible manner. In addition, the study’s findings can help healthcare providers better understand and manage the pain experienced by pediatric patients undergoing dental procedures under GA. This can lead to improved pain management strategies and ultimately better outcomes for pediatric patients. Overall, the study provides valuable information that can contribute to the improvement of pediatric dental care under GA. The study also compared the differences in postoperative discomfort between genders, and found no significant difference. The results showed that restoration was the only variable that indicated discomfort. The study had a limited sample size, however, which may have impacted the results, suggesting the need for larger sample size studies to generalize the findings. Overall, the study contributes to a better understanding of the prevalence, severity, duration, and factors related to postoperative discomfort in young children following GA, which is essential for improving the quality of care provided to these children.

Perception of pain in children is difficult due to their inability to communicate and describe pain [33]. In this study the minimum age of the included participants was 4 years old, since this age category has been proven to be expressive and can converse normally, as mentioned in Atan et al.’s study [34]. Although our study found that more than half of the patients (65.2%) were female, other researchers reported 45% females and 55% males [35]. This difference was thought to have originated from the differences in our sample size, although the objectives of those studies were in accordance with the variables that we assessed in our investigation. The postoperative pain control in our investigation could be the reason for the difference compared to that study, where 80–70% of the patients had local anesthesia [19]. All of the cases in the previously mentioned study underwent nasal intubation, and local anesthetic was used in 82.6% of the participants, as has been reported in a number of other studies [36,37]. In our results there was no significant discomfort found in the participants.

Based on our results, all of the patients were released from the recovery room with discomfort according to the FLACC. A total of 56.5% expressed discomfort by crying steadily, including screams, sobs or expressing frequent complaints in the immediate postoperative period. In previous studies, postoperative pain was also a common finding ranging from 36% and 95%. While some studies reported it as an uncommon finding, this variation may be due to the time of pain assessment, type and quantity of the preformed procedures, and the age of the children [19]. Another study showed that the duration of the procedure was significantly related to postoperative pain. Hence, a long duration means rendering complex treatments and an accumulation of discomfort, which causes the child to feel pain. This has been similarly researched in another one of the studies [23].

In our study, the FLACC scale was used to assess pain because previous studies have demonstrated that this pain assessment scale is valid, easy and convenient to use. Postoperative analgesics were taken by 65.2% of the children. However, in one study 60% of the children took analgesics on day 1 while 33% did not, and their highest pain assessment was mild pain which concludes similar results [38].

Our study revealed 78.3% of the children were eating a regular diet after 3 days and did not need any medications afterwards. El Batawi et al. [39] explained that after dental rehabilitation under GA, children experienced no oral symptoms, healthy gums, and an elimination of problems when they tried to eat regular healthy food (in fact, any type of food).

A study mentioned in literature that we came across investigated similar objectives such as ours [39]. The study has several strengths, including its standardized GA protocol and inclusion criteria. Additionally, the use of a questionnaire to collect data from parents is a convenient and cost-effective method of collecting postoperative data. However, the study also had several limitations. Firstly, the study did not have a control group, making it difficult to compare the results to children who did not receive GA. Secondly, it relied on parental reporting of complications, which may be subject to reporting bias or inaccuracies. Finally, the study did not collect data on the long-term effects of GA on children’s dental health.

GA is a treatment method with intrinsic dangers and special advantages [30]. It is necessary to concentrate more on its safety aspects over a longer period of time [40]. There have been reports of postoperative morbidities ranging from minimal to 90% [30]. Several intraoperative complications have also been reported with its use according to other studies [41,42]. Following instructions and taking part in regular training sessions are required for pediatric dentists to keep their skills up to par, and reduce or completely eradicate the risk of negative outcomes. The majority of pediatric patients describe varying degrees of complaints following dental GA procedures [30]. Several postoperative discomforts have been reported, which differ from one another as assessed in some papers [30]. The most frequent complaint is postoperative pain [30]. A total of 8.2% of children who underwent dental GA in one study displayed some postoperative complications [43]. Adverse events could also sometimes be caused by inexperienced hospital employees or clinicians [44].

Postoperative complaints may be caused by a range of variables, including the type of procedure, the length of the general anesthetic, the use of a double throat pack, the patient’s pre-existing health, the expertise of the provider, and the use of local anesthetic [30]. However, the majority of postoperative complaints subsided quickly, and patients soon returned to their regular routines and normal physical exercise [30].

Parents must watch over the child for 24 h after surgery [45]. Structured postoperative training is provided. Parents are informed about any symptoms they may have experienced, the potential course of events, particularly in the first 24 h following surgery, as well as the recommended diet through this written instruction and, following release of the ward, parents should be advised to administer medications such as paracetamol or ibuprofen for the first 24 h. As long as the child exhibits a definite tolerance to liquids, a dose can be given [44]. The pediatric dentist evaluates whether the healing process is proceeding ordinarily during the follow-up appointment. Over time, parents’ attitudes have shifted in favor of GA being accepted to a greater extent [44]. Dental GA is viewed by parents as a therapy option that improves children’s quality of life [41,42,46,47,48,49].

## 5. Limitations

As far as limitations go, we believe the limited sample size of this study can be mentioned as one of its major flaws. Secondly, the use of the FLACC pain assessment scale and the Dental Discomfort Questionnaire (DDQ-8) may have limitations. The FLACC scale may not accurately assess pain in all children, as pain expression and perception can vary widely among individuals. The DDQ-8 may also be limited by recall bias, as it relies on the child’s memory of pain experienced 3 days after the GA procedure. The study’s design also did not include a control group, which limits the ability to make comparisons and draw definitive conclusions about the effectiveness of pain management strategies used during the GA procedure. In addition, the study only collected data related to the immediate postoperative period and 3 days after the procedure. Pain experienced beyond this time frame may not have been captured, limiting the overall understanding of pain experienced by pediatric patients undergoing dental procedures under GA. However, we would also like to mention that ours is a subset of a few studies that have attempted to analyze postoperative pain of pediatric patients undergoing dental treatment under general anesthesia. As such, the authors wish to emphasize the need for more studies to be undertaken in this regard, to validate ours as well as other studies’ observations.

## 6. Conclusions

In this study, results were significant where the children commonly experienced postoperative symptoms such as pain, crying, and sleepiness after dental rehabilitations under GA; however, this significantly decreased over time. Therefore, none of the variables were predictors for the discomfort of the child. The limited sample size may, however, have had an impact on the results; hence, a larger sample size is required.

## Figures and Tables

**Table 1:** The dental discomfort questionnaire (ddq-8).

Is your child:
Biting things with their back instead of front teeth? Putting away something sweet to eat? Crying during meals? Having problems brushing their upper teeth? Having problems brushing their lower teeth? Having problems chewing? Chewing on one side? Grabbing his/her cheek while eating?

**Table 2:** Descriptive data and characteristics of study of children.

Characteristics		Count	%
Age of the child (years) Mean ± SD (range): 5.43 ± 1.53 (4–9)	4.00	10	43.5%
	5.00	1	4.3%
	6.00	8	34.8%
	7.00	1	4.3%
	8.00	2	8.7%
	9.00	1	4.3%
	Total	23	100.0%
Gender	Female	15	65.2%
	Male	8	34.8%
	Total	23	100.0%
History of chief complaint (Months)	1.00	7	30.4%
	3.00	1	4.3%
	4.00	1	4.3%
	12.00	7	30.4%
	24.00	5	21.7%
	36.00	2	8.7%
	Total	23	100.0%

**Table 3:** Questions asked of caregivers.

Characteristics		N	%
Preoperative			
Previous GA	Yes	5	21.7%
	No	18	78.3%
Analgesic antibiotic	Yes	1	4.3%
	No	22	95.7%
History of pain in months Mean ± SD (range)	12.13 ± 11.65 (0.5–36)		
Intraoperative			
Intubation	Nasal	23	100.0%
LA	Yes	19	82.6%
	No	4	17.4%
Dental procedures Mean ± SD (range)			
Restoration 3.57 ± 2.27 (0–6) Pulpotomy 1.74 ± 1.57 (0–5) Pulpectomy 0.39 ± 0.89 (0–4) Crowns 0.48 ± 1.04 (0–4) SCC 2.96 ± 2.40 (0–8) Extraction 2.26 ± 2.42 (0–9) Space maintainer 0.48 ± 0.79 (0–2)			
Postoperative			
Postoperative analgesic	Yes	15	65.2%
	No	8	34.8%
Length of stay in minutes Mean ± SD (range)	27.83 ± 6.37 (15–40)		
Postoperative after 3 days			
Child is eating a regular diet	Yes	18	78.3%
	No	5	21.7%
Child is on any medication	Yes	5	21.7%
	No	18	78.3%
Child has any other mouth problem	Yes	0	0.0%
	No	23	100.0%

**Table 4:** Pre and post DDQ8 distribution.

		Pre			Post		
		0.00	1.00	2.00	0.00	1.00	2.00
Q1	N	11	9	3	20	2	1
	%	47.8%	39.1%	13.0%	87.0%	8.7%	4.3%
Q2	N	11	8	4	20	3	0
	%	47.8%	34.8%	17.4%	87.0%	13.0%	0.0%
Q3	N	8	9	6	18	4	1
	%	34.8%	39.1%	26.1%	78.3%	17.4%	4.3%
Q4	N	10	8	5	18	4	1
	%	43.5%	34.8%	21.7%	78.3%	17.4%	4.3%
Q5	N	11	8	4	11	11	1
	%	47.8%	34.8%	17.4%	47.8%	47.8%	4.3%
Q6	N	9	9	5	18	5	0
	%	39.1%	39.1%	21.7%	78.3%	21.7%	0
Q7	N	13	7	3	22	1	0
	%	56.5%	30.4%	13.0%	95.7%	4.3%	0
Q8	N	12	7	4	20	3	0
	%	52.2%	30.4%	17.4%	87.0%	13.0%	0

**Table 5:** Distribution of FLACC scores.

Score		*n*	%
Face	0.00	5	21.7%
	1.00	8	34.8%
	2.00	10	43.5%
Legs	0.00	6	26.1%
	1.00	12	52.2%
	2.00	5	21.7%
Activity	0.00	7	30.4%
	1.00	8	34.8%
	2.00	8	34.8%
Cry	0.00	5	21.7%
	1.00	5	21.7%
	2.00	13	56.5%
Consolability	0.00	5	21.7%
	1.00	10	43.5%
	2.00	8	34.8%

**Table 6:** Comparison of pre DDQ, FLACC and post DDQ scores between gender.

Scores	Female			Male		*p*
	Mean		SD	Mean	SD	
PRE DDQ8	6.27		4.18	4.88	4.45	0.418
FLACC Score	5.33		3.42	6.38	3.70	0.415
POST_DDQ8	2.27		2.43	0.87	0.99	0.153
Mann–Whitney U test						
Paired samples statistics pre DDQ8 and post DDQ8						
		Mean	N	Std. deviation	Std. Error mean	*p* ^#^
Pair 1	PRE_DDQ8	5.7826	23	4.23145	0.88232	<0.001
	POST_DDQ8	1.7826	23	2.13108	0.44436	

**Table 7:** Linear regression analysis for discomfort after dental treatment of young children under general anesthesia.

Model		Unstandardized Coefficients		Standardized Coefficients	*t*	*p*
		B	Std. Error	Beta		
	Crowns	−0.999	0.728	−0.299	−1.372	0.198
	SCC	0.744	0.485	0.515	1.534	0.153
	Space maintainer	−1.635	1.010	−0.372	−1.618	0.134
	Post OP Analgesic	0.961	1.690	0.135	0.569	0.581
	Length of stay	0.015	0.137	0.028	0.112	0.913
	Regular diet	−1.078	2.597	−0.131	−0.415	0.686
	Analeptic	−0.321	2.440	−0.039	−0.131	0.898
	Restoration	−0.780	0.350	−0.511	−2.230	0.047
	Pulpotomy	−0.850	0.721	−0.386	−1.180	0.263
	Pulpectomy	−0.604	0.877	−0.155	−0.688	0.505
	Extraction	0.695	0.337	0.484	2.063	0.064

Dependent variable: FLACC score.

**Table 8:** Correlations between different variables.

		PRE_DDQ8	FLACC Score	POST_DDQ8	Age	History of Chief Complaint	Duration	Recovery
PRE_DDQ8	CC	1.000	0.049	0.244	0.353	0.382	0.076	−0.439 *
	*p*		0.826	0.263	0.099	0.072	0.729	0.036
FLACC Score	CC		1.000	−0.054	−0.218	−0.235	−0.107	0.237
	*p*			0.807	0.317	0.280	0.628	0.275
POST_DDQ8	CC			1.000	0.097	0.346	0.261	−0.059
	*p*				0.661	0.106	0.230	0.789
Age	CC				1.000	0.249	−0.042	−0.264
	*p*					0.253	0.850	0.223
History of chief complaint	CC					1.000	0.072	−0.421 *
	*p*						0.742	0.046
Duration	CC						1.000	−0.163
	*p*							0.457
Recovery time	CC							1.000
	*p*							

*. Correlation is significant at the 0.05 level (2-tailed), CC = Correlation Coefficient.

## Data Availability

If requested, the principal author can provide the data statement.
